# Assessing Therapist Competence: Development of a Performance-Based Measure and Its Comparison With a Web-Based Measure

**DOI:** 10.2196/mental.7704

**Published:** 2017-10-31

**Authors:** Zafra Cooper, Helen Doll, Suzanne Bailey-Straebler, Kristin Bohn, Dian de Vries, Rebecca Murphy, Marianne E O'Connor, Christopher G Fairburn

**Affiliations:** ^1^ Department of Psychiatry University of Oxford Oxford United Kingdom; ^2^ Department of Psychiatry Yale School of Medicine New Haven, CT United States; ^3^ Icon Patient Reported Outcomes Oxford United Kingdom

**Keywords:** therapist competence, Web-based knowledge assessment, skill assessment, therapist training outcome, scalable assessment, eating disorders, cognitive-behavioral treatment

## Abstract

**Background:**

Recent research interest in how best to train therapists to deliver psychological treatments has highlighted the need for rigorous, but scalable, means of measuring therapist competence. There are at least two components involved in assessing therapist competence: the assessment of their knowledge of the treatment concerned, including how and when to use its strategies and procedures, and an evaluation of their ability to apply such knowledge skillfully in practice. While the assessment of therapists’ knowledge has the potential to be completed efficiently on the Web, the assessment of skill has generally involved a labor-intensive process carried out by clinicians, and as such, may not be suitable for assessing training outcome in certain circumstances.

**Objectives:**

The aims of this study were to develop and evaluate a role-play–based measure of skill suitable for assessing training outcome and to compare its performance with a highly scalable Web-based measure of applied knowledge.

**Methods:**

Using enhanced cognitive behavioral therapy (CBT-E) for eating disorders as an exemplar, clinical scenarios for role-play assessment were developed and piloted together with a rating scheme for assessing trainee therapists’ performance. These scenarios were evaluated by examining the performance of 93 therapists from different professional backgrounds and at different levels of training in implementing CBT-E. These therapists also completed a previously developed Web-based measure of applied knowledge, and the ability of the Web-based measure to efficiently predict competence on the role-play measure was investigated.

**Results:**

The role-play measure assessed performance at implementing a range of CBT-E procedures. The majority of the therapists rated their performance as moderately or closely resembling their usual clinical performance. Trained raters were able to achieve good-to-excellent reliability for averaged competence, with intraclass correlation coefficients ranging from .653 to 909. The measure was also sensitive to change, with scores being significantly higher after training than before as might be expected (mean difference 0.758, *P*<.001) even when taking account of repeated data (mean difference 0.667, *P*<.001). The major shortcoming of the role-play measure was that it required considerable time and resources. This shortcoming is inherent in the method. Given this, of most interest for assessing training outcome, scores on the Web-based measure efficiently predicted therapist competence, as judged by the role-play measure (with the Web-based measure having a positive predictive value of 77% and specificity of 78%).

**Conclusions:**

The results of this study suggest that while it was feasible and acceptable to assess performance using the newly developed role-play measure, the highly scalable Web-based measure could be used in certain circumstances as a substitute for the more labor-intensive, and hence, more costly role-play method.

## Introduction

Recent research interest in the training of therapists in psychological treatments has highlighted the need for rigorous measures of training outcome, with measures of therapist competence being regarded as of particular importance [1-3]. Competence in this context refers to what has been described as “limited-domain intervention competence” [4], that is, the therapist’s capacity to implement a specific form of treatment to the standard needed for it to achieve its expected effects [5]. Generally, it has been agreed that achieving such competence requires knowledge of the treatment, including how and when to use its strategies and procedures, as well as an ability to apply such knowledge skillfully in practice [6,7]. The assessment of knowledge has generally been regarded as relatively straightforward, because there is a well-studied and documented method for assessing this aspect of competence, usually involving the use of some form of multiple-choice testing [8]. Nevertheless, there are, as yet, few standardized measures for assessing knowledge of the type required to establish therapist competence.

On the other hand, the assessment of skill in delivering a psychological treatment is more complex. A widely used method for assessing the skill of therapists involves the evaluation of the quality of their treatment sessions (ie, therapy quality is being used as an index of therapist competence). Assessing therapy quality requires that treatment sessions be evaluated using a standard procedure [9]. In the field of cognitive behavioral therapy (CBT), for example, treatment sessions are generally rated using the Cognitive Therapy Scale (CTS) [10,11] or its revised version (CTS-R) [12]. Treatment sessions, or usually recordings of them, are evaluated by highly trained raters (usually therapists) with respect to the presence and quality of certain features displayed by the therapist (eg, the eliciting of key cognitions, the use of guided discovery, the setting of homework). Those who score above a prespecified threshold are judged to have performed sufficiently skillfully to be judged competent. This method has the advantage of directly assessing therapists’ skill at implementing the treatment, and thus, has clear ecological validity. In practice, the method poses a number of problems. It is labor-intensive, with the result that few sessions tend to be rated. Consequently, generalizations about the therapist’s overall competence are made on the basis of rating a limited number of the treatment’s procedures. The issue of patient variability is an additional problem. It has been documented that therapists are less likely to adhere to a treatment protocol with some patients rather than others, for example, when comorbidity is present or when they perceive patients’ difficulties to be particularly severe. [13]. Thus, with this method, it is difficult to sample the full range of a treatment’s procedures with patients of varying levels of difficulty [5,6,14]. A related issue is that the CTS and CTS-R, for example, focus largely on aspects of treatment that are common to most forms of CBT and, of necessity, ones that are expected to be present in most treatment sessions. Thus, they assess generic skills but do not assess disorder-specific strategies. In the area of social anxiety disorder, for example, this has led to the development of a disorder-specific measure [15,16].

A potential solution to these problems lies in role-play–based assessments using simulated standardized patients. This method offers the possibility of assessing therapists’ skills on a wide range of procedures or interventions while controlling the variability of the patient. For many years, this type of assessment has formed part of the objective structured clinical examinations used in evaluating medical training [17] and has been described as one of the most effective “substitutes for reality” [6]. The method also lends itself to the evaluation of psychological treatment training, a situation in which the assessment of a range of patient sessions before and after training would otherwise be difficult to achieve. Indeed, this method has just begun to be used to assess skill acquisition following psychological treatment training [18-21].

This study was part of a program of work designed to develop scalable methods for training therapists to deliver evidence-based psychological treatments. It used enhanced cognitive behavior therapy (CBT-E) for eating disorders [22-24] as the exemplar treatment. This treatment is described in detail in a comprehensive treatment guide [25], and an outline of its main stages and procedures is shown in Table 1.

As an essential first step in our work to develop a scalable method of training, we planned to develop methods of assessing training outcome that would also be scalable. Consistent with the view outlined above, we aimed to develop a measure of applied knowledge of CBT-E and a measure of skill at implementing it. We first developed and validated a Web-based measure of applied knowledge of CBT-E [26]. The eMeasure is a brief, scalable, 22-item, Web-based measure for testing applied knowledge of CBT-E, taking about 30 min to complete. It was tested on a relatively large, heterogeneous sample of clinicians at different levels of training. It meets the stringent requirements of the Rasch model and has 3 equivalent versions making it suitable for repeat testing of trainees in outcome studies. Best cut points have been established empirically to distinguish between those judged competent by experts and those who were not.

In this paper, we describe the second stage of the work. This involved developing a performance-based role-play measure of skill at delivering CBT-E to complement the applied knowledge measure. The performance-based measure was designed as a structured role play with therapists being asked to implement a range of CBT-E procedures with a simulated *patient* that would be recorded and subsequently rated for competence. To provide evidence to support the use of this measure, we have described its content and the steps involved in its development. Its performance in assessing clinicians at various stages of training was investigated by examining the feasibility and acceptability of the measure, its sensitivity to change after training, and its interrater reliability. In addition, given our interest in scalability, we also investigated its performance in relation to the previously validated scalable measure of applied knowledge to ascertain whether under certain circumstances it might be possible to use the Web-based measure alone to efficiently assess the outcome of training.

## Methods

### Design

This study was conducted in 3 phases. In the first, clinical scenarios for the role plays were developed and piloted together with a rating scheme for assessing trainees’ performance. In the second, the scenarios were evaluated by examining the performance of a range of trainee therapists from different professional backgrounds and with differing levels of experience in implementing CBT-E. In the third, the relationship between the role-play method of assessing competence and the previously developed Web-based measure of applied knowledge was investigated. Ethical approval was obtained from the Oxford University Central Research Ethics committee.

#### Phase One—The Development of the Clinical Scenarios and Rating Scheme

It was decided in advance that the role-play scenarios should focus on all the main CBT-E procedures and that they should not be inordinately difficult. Each scenario would involve the trainee therapist “treating” a “patient” who would be role-played by an actor (acting as a patient). The scenarios would last no longer than 12 min and 3 different scenarios would be administered in sequence within 1 session, thus enabling the implementation of 3 different procedures to be tested in a 45-min assessment session. We also decided to use 2 different “patients,” each representing a type of patient commonly encountered when helping those with an eating disorder. Patient A was reticent and anxious with an eating style that was generally rigidly controlled, whereas Patient B was talkative and easily distracted and had a more chaotic eating pattern.

The next step was to identify the skills needed to implement CBT-E. To ensure adequate sampling of the potential content, we developed a *blueprint*. When developing assessment measures, blueprints are commonly used to match the elements of the assessment measure to the content to be mastered [27,28]. Using the CBT-E treatment manual [25] as our source, we obtained agreement between the CBT-E treatment developers (CF and ZC) and 3 experienced trial therapists (SBS, KB, and RM) that the role-play measure should focus on the implementation of 10 key procedures. We then developed a *scenario* for each procedure (see Table 1), the goal being to create a partially standardized interaction, that is, one that was focused on the implementation of a particular procedure but not so scripted as to be unrealistic. To this end, we prepared, for the actors, a written account for each scenario describing how the patient should respond in general and the particular patient’s manner of responding, depending upon whether she was Patient A or Patient B. For the person being assessed (ie, the trainee therapist), we prepared for each scenario a written description of the clinical situation to be addressed using CBT-E, a summary of the patient’s progress in treatment so far and some information about her circumstances, personality, and history. To create realistic encounters, we modeled these descriptions on actual patient-therapist interactions. Pilot work led to the 10 clinical scenarios being standardized to 8-min therapist-patient interactions, with each being preceded by 5 min of preparation time for the trainee being assessed.

We decided to use a global scale for rating trainees’ performance rather than a checklist, as global measures have been shown to have greater validity in discriminating levels of expertise in complex interactions [29-31]. Two rating scales were developed, one to assess the quality of implementation of the procedure in terms of its content and the other to assess whether the trainee’s style was consistent with CBT-E. We developed detailed scenario-specific guidelines specifying both core and desirable features to guide the former ratings and a description of CBT style that included details about therapist behavior that would be both appropriate and inappropriate to guide the latter ratings. These guidelines formed the raters’ manual that guided rater training and was available to raters when making their ratings of trainees’ performance.

To rate the quality of implementation of participants’ performance in terms of content, a 7-point scale was developed with defined anchor points. The scale ranged from a complete absence of the relevant CBT-E procedures as specified in the scenario-specific guidelines (score=0) to the consistent and complete application of all these procedures (score=6), with a score of 2 indicating limited or inconsistent application of the core features and a score of 4 indicating moderate application of these features (most of the main features present). Remaining scores were used to indicate performance falling between the defined anchor points. On the basis of our extensive experience in training therapists to implement CBT-E, a rating of 4 or more on this scale (defined as at least moderate application of CBT-E procedures) was taken to represent the cut point for “competent” performance at implementing each procedure. With regard to rating the trainee therapist’s style, raters were provided with a detailed description of generic aspects of CBT style (such as being warm, empathic, collaborative, asking open-ended questions, focusing on and encouraging change) as well as a description of those features that would be inappropriate (such as being insensitive to the patient’s feelings, not attending to the patient’s distress, being critical, lacking professional boundaries, personal disclosures demonstrating behavior inconsistent with the advice given to the patient, being controlling). A yes or no rating assessed whether the trainee therapist adopted a CBT-consistent style.

In practice, the rater first made a procedural rating on the 7-point scale to reflect the quality of implementation of the relevant CBT-E scenario-specific procedure (ie, doing the right thing well). Then if a therapist received a rating of 4 or more (indicating a competent performance on the procedural rating), the rater was required to consider whether an otherwise competent performance was potentially undermined by answering the yes or no categorical question regarding the trainee therapist’s CBT-E style. If this was answered in the affirmative, the rater was required to re-rate the therapists’ performance on the 7-point scale using only a restricted 0 to 3 rating, reflecting the decision that the therapist could not obtain an overall score of 4 or more in the presence of a CBT-E inconsistent style.

**Table 1:** Blueprint showing scenarios for enhanced cognitive behavioral therapy (CBT-E) procedures.

CBT-E treatment stage	Scenario number	Scenario content
Stage 1	1	Creating a case formulation
	2	Reviewing self-monitoring
	3	Implementing and reviewing regular eating
	4^a^	Motivation—encouraging and maintaining engagement in treatment
	5	Collaborative weighing and interpreting weight change
Stage 2^b^		Reviewing progress and planning stage 3
Stage 3	6	Addressing body checking
	7	Addressing residual binges
	8	Addressing avoided foods and dietary rules
	9	Addressing feeling fat
Stage 4	10	Recognizing and manipulating mindsets and preparing for the future

^a^This scenario focuses on a topic relevant to all stages of treatment. The particular situation in the case of Scenario 4 is of a patient in stage 1 of treatment.

^b^Stage 2 in treatment is a transitional stage and does not have an associated scenario.

#### Phase Two—Evaluation of the Clinical Scenarios

To examine the performance of the clinical scenarios, we recruited a sample of 93 therapists who wished to be trained in CBT-E by our group. They were recruited (and participated) at various stages in the training process. Before training, therapists were recruited from the following sources: those registered to attend a conventional 2-day CBT-E training workshop and those about to begin a Web-based training in CBT-E. After training, therapists were recruited from those who had just completed a 2-day workshop, those who had completed a course of expert-led supervision in CBT-E, and those who had completed Web-based training.

Each trainee therapist completed 3 clinical scenarios on any 1 testing occasion selected from the 20 possible scenarios (10 scenarios each with Patient A or B). Two therapists (SBS and KB) role-played the patients. The encounters were audiorecorded for subsequent rating by at least 2 trained raters (see Table 2) who were blind to the identity and training status of the trainee therapists. The trainees were also asked to rate the extent to which they thought their performance in the role plays resembled their usual everyday clinical performance. To do this, a simple 4-point scale (from no resemblance=0 to close resemblance=3) was used. In addition, after completing the role-play assessment, the trainees were asked to complete the Web-based measure of applied knowledge of CBT-E [26] within 2 weeks. The trainees were not provided with any feedback after completing these summative assessments.

Four raters, research assistants with psychology degrees, were trained to assess the trainees’ performance in the role plays. After didactic training in the use of the rating scale provided by 2 experts, the raters were then required to rate 40 prerecorded calibration [32] role plays covering the 10 scenarios and both patients. These had been previously rated by 2 expert clinicians (ZC and RM). Training ratings were compared with the experts’ calibration ratings and any discrepancies discussed and clarified to obtain consensus with the expert calibration ratings before beginning to rate the trainees’ performance on the role-play assessments [15,33].

#### Phase Three—Relationship Between the Web-Based Measure and the Role-Play Measure

The trainee therapists’ scores on the Web-based eMeasure completed at the same time point as their role-play performance (ie, time-matched) were compared with their ratings on the role-play measure to determine whether competence on the former (a scalable measure of applied knowledge of the treatment) predicted competence on the role-play measure (performance skill at applying the treatment).

### Data Analysis

Data were analyzed using SPSS version 19.0 (IBM Corp) and Stata version 12.0, (StataCorp). Both individual performance scores (categorical scores) and scores averaged over all 3 clinical scenarios in any one assessment (continuous scores) were used in the analyses. Average performance scores on the clinical scenarios were approximately normally distributed. Values of n (%), mean (SD), and median (range) were used as appropriate to describe the data, with chi-square tests (with linear trend where appropriate) used to compare categorical scores between groups and *t* tests and ANOVAs (analyses of variance) and linear mixed models to compare continuous scores.

Agreement between categorical individual performance scores given by each of the raters was assessed using kappa statistics with Cohen kappa statistics used to assess agreement between pairs of raters and Fleiss kappas to calculate agreement across all 4 raters. Analyses were conducted first using all possible rating scores (ie, scores 0-6) and then on binary variables defined as scores over the threshold for competence (ie, 4 or more). Values of kappa <0 were taken to indicate no agreement, 0 to 0.20 slight, 0.21 to 0.40 fair, 0.41 to 0.60 moderate, 0.61 to 0.80 substantial, and 0.81 to 1 almost perfect agreement over that expected by chance [34].

Agreement between continuous scores was assessed with intraclass correlation coefficients (ICCs). With the average performance scores, ICCs (2-way random effects model, ie, ICC2,1 for single measures and ICC2,k for average measures) were calculated between raters in pairs, in triplets, and over all 4 raters, with values of <0.4 indicating poor agreement, 0.4 to 0.75 fair-to-good agreement, and >0.75 excellent agreement [35].

To assess the impact of time (before or after training), rater, simulated patient type, and particular scenario on performance scores, data were first analyzed using ANOVA to compare performance scores between groups. An interaction term between rater and time was also fitted. Values of eta-squared (*η*^2^) were used to express the percentage of variance explained by each factor, with 0.02 considered to be small, 0.13 medium, and 0.26 large [36]. To take account of the repeated nature of the data and the specific correlation structure of nonindependent (repeated) data, linear mixed models with variance components were used. This is a form of Generalizability theory (G-theory) in which the sources of measurement error are identified, estimated, and disentangled [37]. Models were fitted with fixed and random effects as appropriate for time, rater, time × rater, patient type, and scenario. The relative contribution of these factors to the variability in the model was estimated using the ICC, calculated as the variance of each individual effect divided by the overall variance.

To determine whether competence on the Web-based eMeasure predicted competence on the role-play measure, the competence scores of each trainee therapist (average performance score over all raters on each occasion on the clinical scenarios [averaged over 3 scenarios] rated as >4) and their competence score on the Web-based eMeasure (using the previously reported cut points for competence [26]) were matched. Values for the sensitivity, specificity, positive predictive value (PPV), and negative predictive value (NPV) of the Web-based measure were calculated.

## Results

### Characteristics of the Trainee Therapists

The mean age of the 93 trainee therapists who took part in the role-play based assessment was 42.7 (SD 9.6) years, and the majority (77 trainees) were female (83%). Their mean number of years of clinical experience was 15.0 (SD 9.65) with their professional backgrounds being as follows: 33 clinical psychologists (35%); 26 psychiatric nurses or nurse therapists (28%); 7 eating disorder therapists (8%); and 4 occupational therapists (4%). The remaining 23 trainee therapists (25%) came from a variety of other professional groups, including social workers, psychiatrists, and dietitians. Eighty-seven trainees (94%) encountered patients with eating disorders in their clinical practice, with 65 (75%) doing so regularly. A substantial majority of the trainees (78/93, 90%) also treated patients with eating disorders.

### Scenario and eMeasure Completion

Of the 93 trainee therapists, there were 27 in the workshop-only training group, 36 in the workshop plus clinical supervision group, and 30 in the Web-based training. All the trainees completed at least one set of (3 different) scenarios, either before or after receiving training, with 24 therapists completing them before training only, 29 after training only, and 40 therapists completing scenarios both before and after training. Before training, 64 (24+40) trainees completed a set of scenarios and an accompanying eMeasure (both completed at the same time point, ie, time-matched), whereas after training 68 had a complete set of time-matched role-play and accompanying eMeasure score (1 eMeasure score was lost because of technical failure: 29+40−1). Results are reported separately for groups before training and after training.

Of those who treated patients, 60 trainees (77%) rated that their performance moderately, or closely (a rating of 2 or 3 on the 0-3 scale), resembled their usual clinical performance.

### Agreement Between the Raters

Overall, ratings of the mean Cohen kappa statistic for the binary ratings of competence (averaged over the 3 scenarios) were moderate, with a kappa of 0.51 over pairs of raters and with a Fleiss combined kappa of 0.52.

ICCs assessing the reliability of the raters’ scores, assuming a single rater or average scores for more than 1 rater (as was the case for all competence ratings in this study), are shown in Table 2 (data include ratings given both before and after training). The mean (standard error, SE) scenario scores for each rater (rated on the 7-point rating scale) are shown along with the ICCs for each combination of raters, indicating fair-to-good agreement in all cases and excellent agreement in the majority of cases of the average ratings.

### Factors Contributing to Variance in Performance Scores

Performance scores increased significantly from before to after training (mean difference 0.758, *P*<.001; effect size 0.51). After taking the repeated nature of the data into account, the adjusted mean difference remained highly significant (mean difference 0.667, *P*<.001) but with a smaller effect size at 0.33 (see Table 3).

Before training, there was no significant difference in the mean rating scores of the 4 raters (*P*=.56, *η*^2^=0.005). Although there was a significant difference after training (*P*=.01), the value of *η*^2^was small (0.020). Although the main effect of rater was nonsignificant (*P*=.65), there was an overall effect of time (mean difference: 0.80, SE=0.091, *P*<.001) and a significant interaction between rater and time (*P*=.02). In the mixed model analyses adjusting for repeated data, the significant effect of time remained (mean difference: 0.79, SE=0.117, *P*<.001), and there was a significant interaction between time and rater (*P*=.03) but no significant overall effect of rater (*P*=.16).

Using mixed model (variance components) analyses with fixed effects for time, patient type, and scenario, and random effects for trainee and rater, there was no significant effect of patient type (Patient A or B; *P*=.41), but there was a significant effect for scenario (*P*<.001), with 5 scenarios (scenarios 1, 2, 5, 9, and 10) given significantly lower ratings than the other 5 (mean difference: −0.48, SE=0.088), thus suggesting that they were more challenging than the others. Trainee therapists were, however, just as likely to receive these scenarios as the other potentially easier scenarios (with 505/984, 51.3% of scenario scores derived from the 5 less challenging ones).

**Table 2:** Mean scenario scores and values of intraclass correlation coefficient (ICC) for agreement between groups of raters.

Raters	N	Mean (SE^a^)				Single ICC2,1^b^	Average ICC2,k^b^
		Rater 1	Rater 2	Rater 3	Rater 4		
1, 2, 3, 4	15	4.03 (0.35)	4.01 (0.27)	3.31 (0.33)	3.88 (0.43)	0.653	0.883
1, 3, 4	15	4.03 (0.35)	-	3.31 (0.33)	3.88 (0.43)	0.694	0.872
1, 2, 3	28	3.90 (0.23)	3.87 (0.18)	3.58 (0.22)	-	0.616	0.828
1, 2, 4	22	3.64 (0.29)	3.67 (0.23)	-	3.85 (0.34)	0.619	0.830
2, 3, 4	15	-	4.01 (0.27)	3.31 (0.33)	3.88 (0.43)	0.620	0.830
1, 2	54	3.48 (0.16)	3.47 (0.13)	-	-	0.833	0.909
1, 3	28	3.90 (0.23)	-	3.58 (0.22)	-	0.517	0.682
1, 4	22	3.64 (0.29)	-	-	3.85 (0.34)	0.604	0.753
2, 3	28		3.87 (0.18)	3.58 (0.22)		0.542	0.703
2, 4	22	-	3.67 (0.23)	-	3.85 (0.34)	0.485	0.653
3, 4	96	-	-	3.50 (0.11)	3.67 (0.12)	0.690	0.816

^a^SE: standard error.

^b^ICC: intraclass correlation coefficient.

**Table 3:** Mean (standard error) and median scores of trainee therapists before and after training.

Scenario scores		Mean (SE)^a^	Median	Mean difference (SE)	Effect size	P value (*η*^2^)
**Scenario scores unadjusted for repeated data**						
	Before training (N=411)	3.15 (0.073)	3.0			
	After training (N=573)	3.90 (0.056)	4.0	0.758 (0.090)	0.51	<.001 (0.067)
**Scenario scores adjusted for repeated data^b^**						
	Before training (N=411)	3.22 (0.10)				
	After training (N=573)	3.89 (0.10)		0.667 (0.10)	0.33	<.001

^a^SE: standard error.

^b^Fixed effects=time; random effects=therapist trainee.

**Table 4:** Number of trainee therapists achieving competence as assessed by the skill (role-play) measure and the eMeasure.

Competence	Before training			After training		
	Skill (role-play) measure					
eMeasure	Competent, n	Not competent, n	Total, N	Competent, n	Not competent, n	Total, N
Competent, n	2	0	2	24	7	31
Not competent, n	19	43	62	12	25	37
Total, N	21	43	64	36	32	68

### Relationship Between the Web-Based Measure and the Role-Play Measure

The ability of the Web-based measure (eMeasure), with an effect size of 0.46 for change following training, to predict competence on the role-play measure was assessed. The findings are shown in Table 4. Before training (64 trainee therapists), the sensitivity of the Web-based measure was 10% (2/21), whereas after training (68 trainee therapists) it was 67% (24/36). The figures for specificity were 100% (43/43) and 78% (25/32), respectively. NPV of the Web-based measure was 69% (43/62) before training and 68% (25/37) after training, and PPVs were 100% (2/2) and 77% (24/31), respectively. Thus, the majority of trainee therapists who were judged competent on the Web-based measure were also judged competent on the role-play measure.

## Discussion

### Principal Findings

There are at least two components involved in assessing therapist competence: the assessment of their knowledge of the treatment concerned, including how and when to use its strategies and procedures, and an evaluation of their ability to apply such knowledge skillfully in practice. Using CBT-E as an exemplar, this paper describes the development and the evaluation of the performance of a role-play based measure of skill at delivering CBT-E designed as a complement to the previously developed and validated Web-based measure of applied knowledge [26]. It also reports results of a comparison of the performance of these measures at assessing therapist competence with particular emphasis on whether the more scalable Web-based measure was able to predict performance on the more time-consuming and complex role-play measure.

The role-play based measure had a number of strengths. It assessed actual performance skill rather than mere knowledge and understanding; it was possible to test trainee therapists’ performance on a range of key CBT-E procedures; and it did this within an hour. Furthermore, it was found that trained raters could rate trainees’ performance with moderate-to-good agreement for binary ratings of competence between pairs of raters, and more importantly, good-to-excellent reliability for averaged competence. The measure was also sensitive to change, with scores being significantly higher after training than before, as might be expected. Of note, as well, the majority of the trainee therapists thought that their role-play performance resembled their everyday clinical practice. A further potential advantage is that the role-play measure could be used as a formative assessment to assess and improve performance during training. In the present context, it was used purely as a summative assessment measure, but it is clear that its design does not preclude its use as a formative assessment tool.

The major shortcoming of the role-play–based measure was that it required considerable time and resources. This shortcoming is inherent in the method. Although the medical literature has long recognized that standardized patient evaluations are a good substitute for reality [6], they have also been recognized as complex to devise and expensive to implement [17], especially when the need to devise relatively long and comprehensive assessments is taken into account [14].

The relationship between the scores on the Web-based measure of applied knowledge and those on the role-play measure is therefore of importance, given the ease of use and scalability of the former. It was found that the Web-based measure had a PPV of 77% after training. Thus, the majority of those judged competent on the Web-based measure were also found to be competent on the role-play measure. As role-play tasks are generally accepted as simulating reality well and the therapists regarded their performance as being representative of their clinical practice, it seems reasonable to take estimates of competence on the Web-based measure as a good substitute for a skill-based measure.

### Limitations

Some limitations of this work need to be recognized. First, despite recruiting almost 100 trainee therapists to help evaluate the performance-based measure, a larger sample might have allowed further refinement of the role-play measure by allowing reassessment of its performance after modifications. Ideally, all scenarios would be of equal difficulty or the difficulty level of the scenarios could be better ranked for use in further competence testing. Larger numbers of trainees tested would also have potentially strengthened confidence in the results of the comparison of trainees’ performance on the 2 measures. Second, although cut points for competence by their very nature require expert judgments, it would have been preferable to validate these against treatment outcome. However, obtaining such data presents considerable practical obstacles. Third, we have used just one treatment as an exemplar. It cannot be assumed that similar findings would be obtained with all psychological treatments. Fourth, while establishing competence at a given time (eg, passing a driving test) may make ongoing high-quality performance both feasible and more likely, it does not ensure it (consistent high-quality driving). Thus, there is a need to complement the assessment of competence, required for training outcome, with the assessment of the quality of therapists’ performance over time. This is especially important to combat the well-documented phenomenon of “therapist drift” [38,13]. Finally, whereas the cost of an easily administered Web-based measure that can be scored automatically is likely to be less than more traditional and labor-intensive method of assessing competence, a formal study of cost-effectiveness was unfortunately beyond the scope of this work.

### Conclusions

In summary, this study describes the development and testing of a performance-based measure of skill at delivering CBT-E. Although the measure performed reasonably well, it had inherent disadvantages in terms of scalability. It is therefore of considerable interest that the Web-based measure of applied knowledge of CBT-E was relatively efficient at predicting competence as assessed by the role-play measure. This indicates that the scalable Web-based measure could be used in certain circumstances to assess the outcome of training in CBT-E.

## Abbreviations

ANOVA: analysis of variance
CBT: cognitive behavioral therapy
CBT-E: enhanced cognitive behavioral therapy
CTS: Cognitive Therapy Scale
CTS-R: revised Cognitive Therapy Scale
ICCs: intraclass correlation coefficients
NPV: negative predictive value
PPV: positive predictive value
SD: standard deviation
SE: standard error
